# AQUA Cloning: A Versatile and Simple Enzyme-Free Cloning Approach

**DOI:** 10.1371/journal.pone.0137652

**Published:** 2015-09-11

**Authors:** Hannes M. Beyer, Patrick Gonschorek, Sophia L. Samodelov, Matthias Meier, Wilfried Weber, Matias D. Zurbriggen

**Affiliations:** 1 Faculty of Biology, University of Freiburg, Freiburg, Germany; 2 Spemann Graduate School of Biology and Medicine (SGBM), University of Freiburg, Freiburg, Germany; 3 BIOSS Centre for Biological Signalling Studies, University of Freiburg, Freiburg, Germany; 4 IMTEK, Department of Microsystems Engineering, University of Freiburg, Freiburg, Germany; Indian Institute of Science, INDIA

## Abstract

Assembly cloning is increasingly replacing conventional restriction enzyme and DNA-ligase-dependent cloning methods for reasons of efficiency and performance. Here, we describe AQUA (advanced quick assembly), a simple and versatile seamless assembly cloning approach. We demonstrate the applicability and versatility of AQUA Cloning in selected proof-of-principle applications including targeted insertion-, deletion- and site-directed point-mutagenesis, and combinatorial cloning. Furthermore, we show the one pot *de novo* assembly of multiple DNA fragments into a single circular plasmid encoding a complex light- and chemically-regulated Boolean A NIMPLY B logic operation. AQUA Cloning harnesses intrinsic *in vivo* processing of linear DNA fragments with short regions of homology of 16 to 32 bp mediated by *Escherichia coli*. It does not require any kits, enzymes or preparations of reagents and is the simplest assembly cloning protocol to date.

## Introduction

The emerging field of synthetic biology seeks for efficient ways to engineer novel genetic circuits and switches. Inspired by electrical engineering, which combines well-specified and characterized electronic elements to construct devices with defined performance, modern synthetic biological engineering aims to follow the same principles of rational design, simulation, construction and verification [1,2].

The molecular toolbox required for such design approaches must include suitable cloning methods for convenient and reliable assembly of functional biological elements on a genetic level. While conventional type II restriction enzyme- and DNA-ligase-dependent cloning has provided valuable possibilities for the manufacturing of recombinant genetic material in the past, more avant-garde techniques recently overcame inherent drawbacks of conventional cloning, such as the introduction of seams, or the limited availability of unique restriction enzyme sites. Such seamless assembly cloning methods are generally based on *in vitro* or *in vivo* recombination events of short homologous regions flanking double-, or partially single-stranded linear DNA molecules and allow for a one-pot, one-step assembly of multiple DNA fragments to form a desired circular plasmid. Gibson assembly [3], ligase cycling reaction [4], Restriction-free cloning [5] and In-Fusion assembly [6] are prominent examples of *in vitro* methods relying on recombinant thermo-stable enzymes, such as exonucleases, polymerases and ligases. Other methods, including circular polymerase extension cloning [7], USER [8] and SLiCE [9] are semi-*in vitro*, resulting either in enzymatically non-covalently closed plasmids that are finalized *in vivo* by *E*. *coli* or are based on bacterial lysates prepared from recombinase-engineered strains. Enzyme-free cloning [10–12] is another semi-*in vitro* method that requires double pairs of PCR primers along with additional steps for heteroduplex formation of the amplified DNA molecules. Finally, yeast homologous recombination [13] allows the *de novo* generation of circular plasmids from DNA fragments sharing overlapping homologous regions, yet is only compatible with the few commonly available plasmids, harbouring an appropriate origin of replication.

Current assembly cloning methods excel at versatility and efficiency, yet also suffer from a labor- and cost-intensive process of establishment, optimization and preparation, which is accompanied by the underlying experimental complexity. In this work, we describe a cost-efficient and simple cloning approach, AQUA (**a**dvanced **qu**ick **a**ssembly), and demonstrate its versatility in selected proof-of-principle applications for molecular and synthetic biology systems in bacterial, mammalian and plant cells. The field of application ranges from (multiple) DNA fragment assembly, insertion- and deletion-mutagenesis, combinatorial cloning, the quick coupled cloning and protein expression in the bacterial expression host cells (AQUA Expression), up to the introduction of point-mutations into target sequences. This DNA assembly method relies on *in vivo* processing by *E*. *coli* and allows any molecular biology laboratory to instantly go for seamless standard and sophisticated cloning approaches without any need of establishment or the purchasing of kits, chemicals, cell preparations or additional enzymes. DNA parts are prepared either by PCR, or by restriction digest (or both), sharing 16 to 32 bp of homologous sequence with each adjacent DNA fragment. This principle of *in vivo* cloning in *E*. *coli* has raised only little attention in the past, but it is just gaining momentum as described recently [14]. AQUA Cloning facilitates powerful multi-part assembly at low-cost and in a quick and convenient work-flow. AQUA Cloning is both simple and reliably usable in ordinary lab strains of *E*. *coli* as demonstrated here in experimental examples covering common tasks of a modern biologist as well as for the generation of a sophisticated light- and chemically-responsive synthetic Boolean operation encoded in a single plasmid.

## Methods

### Plasmids and oligonucleotides used in this study

All plasmids and oligonucleotides generated or used in this study are described in Table A in S1 File.

### Preparation of DNA

DNA fragments were generated either by PCR, or by restriction digest. PCR amplification was performed using 1 μL DNA template (50–100 ng), 10 μL Q5 Reaction Buffer (NEB), 4 μL dNTPs (2.5 mM), 1 μL DMSO, 0.5 μL Q5 High-Fidelity DNA polymerase (NEB), 1 μL reverse primer (10 μM) and 1 μL forward primer (10 μM), filled up to a total volume of 50 μL with 31.5 μL ddH_2_O. DNA oligonucleotides were designed with melting temperatures for the annealing sequences of 60°C according to SantaLucia [15] as determined with Genious R7 (Biomatters). For amplification, a 20 cycles (step 2–4) PCR program with 5 min / 98°C, 30 s / 90°C, 30 s / 60°C, 40 s/kb / 72°C and 10 min / 72°C was used. PCR products were separated by gel electrophoreses on 1–2% agarose gels with 1 μg/mL ethidium bromide in 0.5x TAE buffer. For gel extraction the QIAquick Gel Extraction Kit (QIAGEN) was used according to the instructions of the manufacturer. The DNA was eluted in 22 μL ddH_2_O. Typically, yields of 20-80 ng/μL were obtained as quantified using a Nano Drop 1000 Spectrophotometer (PEQLAB Biotechnologie GmbH). For restriction digest, 40 μL DNA (2–4 μg) were mixed with 2.5 μL of each enzyme (NEB), and 5 μL of the corresponding 10x Buffer (NEB). The samples were digested at 37°C overnight. Linearized DNA was gel-purified as described for PCR products.

### AQUA Cloning

For AQUA Cloning, all DNA fragments were mixed in a total volume of 10 μL ddH_2_O with molar ratios of 3:1 (insert:vector) and 12 ng of linearized vector per 1 kb vector size. DNA mixtures were incubated for 1 h at the indicated temperatures (room temperature [RT] unless stated otherwise) and were subsequently transformed into a 25 μL aliquot of chemically competent *E*. *coli* TOP10 cells (in-house prepared, competency: 0.6 x 10^7^ colony forming unit (CFU)/μg DNA). Chemically competent *E*. *coli* cells were thawed on ice and 5 μL prepared AQUA DNA mixture was added. Cells were incubated with the DNA for 10 min on ice and a heat shock was performed for 45 s at 42°C. After 2 min of incubation on ice, 250 μL LB medium was added to the cells and they were shaken for 1 h, 37°C, 700 r.p.m. Finally, the cells were spread on LB agar plates containing appropriate antibiotics. Single colonies were selected after 16 h and were analyzed for correct assembly either by restriction digest, or by analytical colony PCR using the Taq DNA polymerase (NEB) according to the instructions provided by the manufacturer. Single clones were confirmed for correct assembly first by analytical PCR or restriction digest and/or by comprehensive sequencing or experimentally.

For additional guidance see Table 1 “Troubleshooting guide for AQUA Cloning”.

**Table 1:** Troubleshooting guide for AQUA Cloning. The issues listed below are based on the experience in our lab.

No	Issue	Cause	Solution
1	No colonies obtained	Cells are not competent or non ideal incubation conditions	Test for competency. Treat cells carefully. Avoid pipetting of cells. Confirm temperature and time for heat-shock.
		Wrong selection pressure was applied.	Confirm antibiotic resistance.
2	Only very few transformants grew.	A high number of DNA fragments is assembled. Also see issue 1.	Double the total amount of DNA and/or increase the amount of competent cells. Vary transformation conditions.
		The product may be toxic.	Incubate cells at lower temperature (20–25°C).
		The DNA fragments are too large.	Try different strains, reduce PCR product size (split), or consider electroporation.
3	Very few clones mediated a correct DNA fragment assembly.	PCR product of low purity.	Always gel-purify the PCR product and cut bands narrow to exclude side-products.
		PCR product is low in yield.	Optimize the PCR reaction or double the reaction volume.
		PCR product concentration is too high.	Eventually dilute the PCR product with water to avoid pipetting of very small volumes.
4	The total volume of 10 μL cannot be met.	The concentrations of PCR products are too low.	Improve PCR reaction protocol or try concentrating the DNA mixture at 60°C for 10 minutes using a heat block. Increase the volume, but also the volume of competent cells.
5	A high number of fragments are required and are troublesome.	The number of DNA fragments must be reduced.	Consider fusion PCR.

### Preparation of chemically competent *E*. *coli* cells

Except when indicated otherwise, AQUA Cloning was performed in the TOP10 (Invitrogen) *E*. *coli* strain which was prepared for competency using the following protocol: 20 mL LB-medium were inoculated in a 100 mL sterile Erlenmeyer flask and were incubated overnight at 37°C, 300 r.p.m. Next day, the pre-culture was used to inoculate four 500 mL sterile Erlenmeyer flasks, each containing 250 mL LB-medium which were incubated at 37°C, 250 r.p.m, until OD_600_ = 0.5–0.6 was reached. Cultures were cooled down on ice for 5 min and were next harvested by centrifugation in sterile vessels at 4°C for 15 min at 4,000 *g*. The cell pellets were resuspended carefully in a total volume of 80 mL of freshly prepared (from stock solutions) ice-cold TFB1 buffer (34.4 mL ddH_2_O, 8 mL 300 mM potassium acetate pH 6.0 (adjusted with KOH), 1.6 mL 500 mM CaCl_2_, 8 mL 500 mM MnCl_2_, 16 mL 500 mM RbCl, 12 mL glycerol, sterile filtered). Cells were centrifuged for 15 min at 4°C at 2,000 *g* and the supernatant was discarded. The cells were then resuspended in a total volume of 8 mL of freshly prepared (from stock solutions) ice-cold TFB2 buffer (4.72 mL ddH_2_O, 0.8 mL 100 mM MOPS pH 7.0, 1.2 mL 500 mM CaCl_2_, 80 μL 1M NaCl, 1.2 mL glycerol, sterile filtered) and were incubated on ice for 15 min. Aliquots of 25 μL were prepared in sterile 1.5 mL Eppendorf tubes and were shock frozen in liquid nitrogen. Competent cells were stored at -80°C for further use. Competency was determined by transformation with the plasmid pSW209 (6,932 bp) typically yielding approximately 10^7^ CFU/μg.

### *E*. *coli* strains

The following commercially available strains of *E*. *coli* were also used for AQUA Cloning: One Shot TOP10 (Invitrogen cat. No. C4040-03, competency: >10^9^ CFU/μg), NEB5α (NEB, cat. No. C2987I, competency: 1–3 x 10^9^ CFU/μg), NEB10β (NEB, cat. No. C3019I, competency: 1–3 x 10^9^ CFU/μg), BL21 (DE3) (NEB, cat. No. C2527I, competency: 1–5 x 10^7^ CFU/μg), JM109 (Promega, cat. No. L2005, competency: 10^8^ CFU/μg).

### Confocal imaging

For confocal imaging, human embryonic kidney cells (HEK-293T) [16], or NIH/3T3 fibroblasts cells (ATCC CRL-1658) were maintained in Dulbecco’s modified Eagle’s medium (PAN, cat. No. P03-0710) supplemented with 10% fetal bovine serum (PAN, cat. no. 1502-P123002, batch no. P123002), 100 U/mL penicillin and 0.1 mg/mL streptomycin (PAN). 50,000 cells were seeded on glass-coverslips and transfected using polyethyleneimine (PEI, linear, MW: 25 kDa, Polyscience) as described elsewhere [17]. The next day, cells were washed once with ice cold Dulbecco's Phosphate-Buffered Saline with Ca^2+^/Mg^2+^ (PAN) and were subsequently fixed with 4% paraformaldehyde for 10 min on ice followed by 10 min at room temperature. Cells were DAPI stained for 10 s in an aqueous DAPI solution (Roth, 1:5,000 dilution of 1 mg/mL stock) and were washed with H_2_O. Coverslips were embedded in Mowiol 4–88 (Roth) containing 15 mg/mL 1,4-diazabicyclo[2.2.2]octane (DABCO, Roth) and were mounted on object slides. Samples were imaged on a confocal microscope system (Nikon Instruments Eclipse Ni-E with a C2 confocal laser scanner, 100x oil objective NA = 1.45). mCherry, mEGFP and DAPI were visualized using excitation lasers of 561 nm, 488 nm, 408 nm, and emission filters of 570–1,000 nm, 500–550 nm, 417–477 nm, respectively. Image acquisition, analysis and processing (brightness and contrast) were performed with NIS-Elements Viewer (Nikon Instruments, version 4.20.00).

### Expression and purification of fluorescent proteins from *E*. *coli*

Bacterial expression vectors were transformed into the *E*. *coli* BL21 (DE3) pLysS strain and grown overnight in LB-medium containing 100 μg/mL ampicillin and 36 μg/mL chloramphenicol at 37°C, 150 r.p.m. On the next day, 1 L cultures were inoculated to a density of OD_600_ = 0.1 and protein expression was induced at OD_600_ = 0.8 at 11°C with 1 mM IPTG overnight. Cultures were harvested by centrifugation at 6,000 *g* for 10 min, 4°C and in the following resuspended in lysis-buffer (50 mM NaH_2_PO_4_, 300 mM NaCl, 10 mM imidazole, pH 8.0). Cell samples were lysed by sonication (Bandleine Sonoplus HD3100, 2 x 10 min with 60% amplitude and 0.5 s / 1 s pulses, invert in between) and the resulting crude lysates were cleared by centrifugation (30,000 *g*, 30 min, 4°C). Hexahistidine-tagged proteins were purified by Ni^2+^-NTA affinity chromatography with 2 mL agarose bead volume per 1 L of expression culture according to the protocol of the manufacturer (Qiagen). Fluorescence was visualized by illumination with 366 nm UV light. Absorbance and fluorescence emission spectra were determined using an infinite M200 pro microplate reader (Tecan). Fluorescence excitation wavelengths were 440 nm for EGFP and 360 nm for EGFP-Y66H.

### Auxin-mediated protein degradation in protoplasts

Protoplast isolation, transformation and auxin treatment were performed as described previously [18]. In brief, protoplasts were isolated from *Arabidopsis thaliana* by plasmolysis and flotation. For each sample, 10^5^ protoplasts were transformed with 20 μg of plasmid DNA using a PEG1500-based method with a total volume of 120 μL of protoplast/DNA mixture and the addition of 120 μL PEG1500-Ca(NO_3_)_2_ solution. Volumes were adjusted to 2 mL with PCA regeneration medium after transformation and protoplasts were incubated in the dark for 24 h. Auxin (indole-3-acetic acid, Sigma, cat. no. 128869) was dissolved in PCA medium and added to a final concentration of 1 μM where appropriate and renilla and firefly bioluminescence was determined after 1 h simultaneously using a BioTec Synergy 4 and Tecan infinite M200 pro plate reader, respectively.

### AQUA Expression

For combined cloning of plasmids and subsequent expression of proteins thereof, AQUA Cloning was performed using 25 μL of BL21 (DE3), or TOP10 *E*. *coli* cells as a control, as described above. After transformation, cells were grown overnight in 10 mL LB-medium containing 50 μg/μL spectinomycin at 37°C, 150 r.p.m. The next morning, the overnight culture was used to inoculate 200 mL fresh, antibiotic-containing LB-medium which was induced with 1 mM IPTG when OD_600_ = 0.9 was reached at 37°C, 150 r.p.m. Cells were harvested 6 h post induction by centrifugation at 5,000 *g* for 5 min, washed with PBS and resuspended in 2 mL PBS before an image was acquired.

### Analysis of light-induced reporter expression in mammalian cells

Chinese hamster ovary cells (CHO-K1, ATTC CCL 61) were maintained in HTS medium (Cell Culture Technologies) supplemented with 10% fetal bovine serum (PAN, cat. no. P30-3602, batch no. P101003TC) and 100 U/mL penicillin and 0.1 mg/mL streptomycin (PAN). 70,000 cells were seeded per well of a 24-well plate one day prior to transfection. Cells were transfected with the bicistronic plasmid pHB368 and the SEAP reporter plasmid pKM006 using PEI (see above). Additionally, the cell culture medium was supplemented with 2 μg/mL tetracycline where appropriate. On the next day, the cells were illuminated with an UV-B narrow-band lamp (Philips, prod. no. PL-S 9W/01) with an intensity of 2.7 μmol/(m^2^•s) as described previously [17], or were kept in darkness. After 24 h of illumination, SEAP reporter activity was determined as described elsewhere [19].

## Results

**Fig 1:**
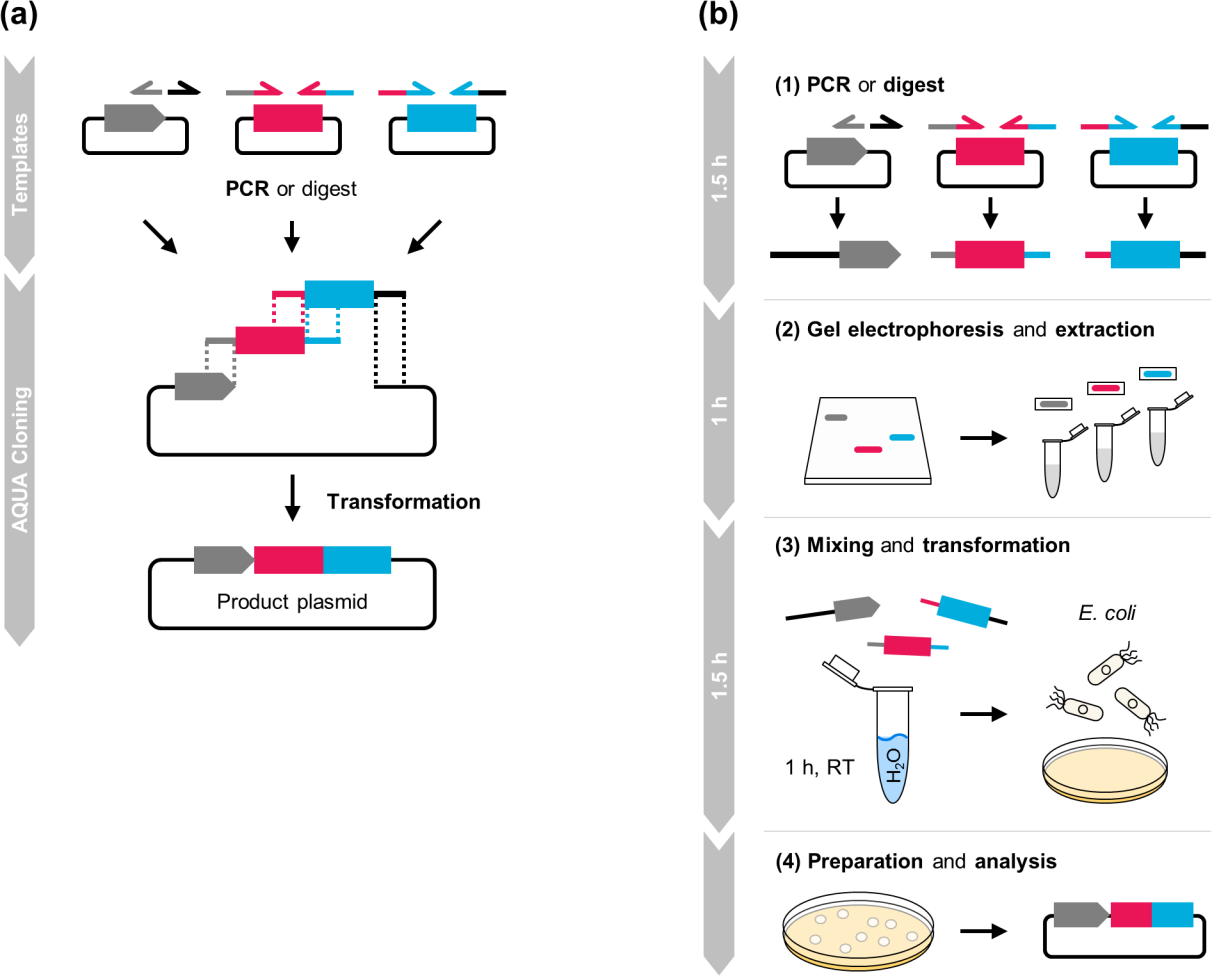
AQUA Cloning: advanced quick assembly cloning. **(a)** DNA parts are produced by PCR, or restriction digest (or both). Oligonucleotides are designed to contribute flanking homologous regions to adjacent DNA fragments of optimally 32 bp in length. DNA parts are assembled into a circular plasmid by sequence-determined directionality. **(b)** AQUA Cloning work-flow. (1) DNA parts are generated by PCR amplification, or derived from an enzymatic digestion. (2) Next, DNA parts are purified by gel-electrophoresis and (3) mixed and simply incubated in water prior to transformation into chemically competent *E*. *coli* Top10 cells for *in vivo* assembly. (4) Finally, obtained colonies are confirmed for correct assembly by standard methods such as analytical PCR, restriction digest, or comprehensive sequencing.

In this work, we introduce AQUA Cloning, a simple and versatile molecular cloning approach. While seamless assembly cloning protocols are available, these methods entail intrinsic requirements of complex reagents such as kits, enzyme and buffer components. AQUA Cloning aims to simplify the cloning procedure by transferring the DNA fragment assembly reaction from an *in vitro* environment into living bacteria and benefits from their inherent *in vivo* enzyme processing activities (Fig 1A and Fig 1B). We first describe the AQUA principles and investigate on the optimal cloning conditions for the construction of two and four DNA fragment assemblies. Next, we show its usage for various commonly needed applications in molecular and synthetic biology laboratories, covering bacterial, mammalian and plant experimental platforms. The examples range from insertion, deletion and targeted point-mutagenesis, combinatorial cloning, the combined cloning and protein expression in a single cell, up to the *de novo* construction of a complex six part, multi-fragment assembly for light- and chemically-controlled Boolean operations, encoded in a single plasmid.

### AQUA Cloning conditions

#### De novo assembly of 2-DNA fragments

**Fig 2:**
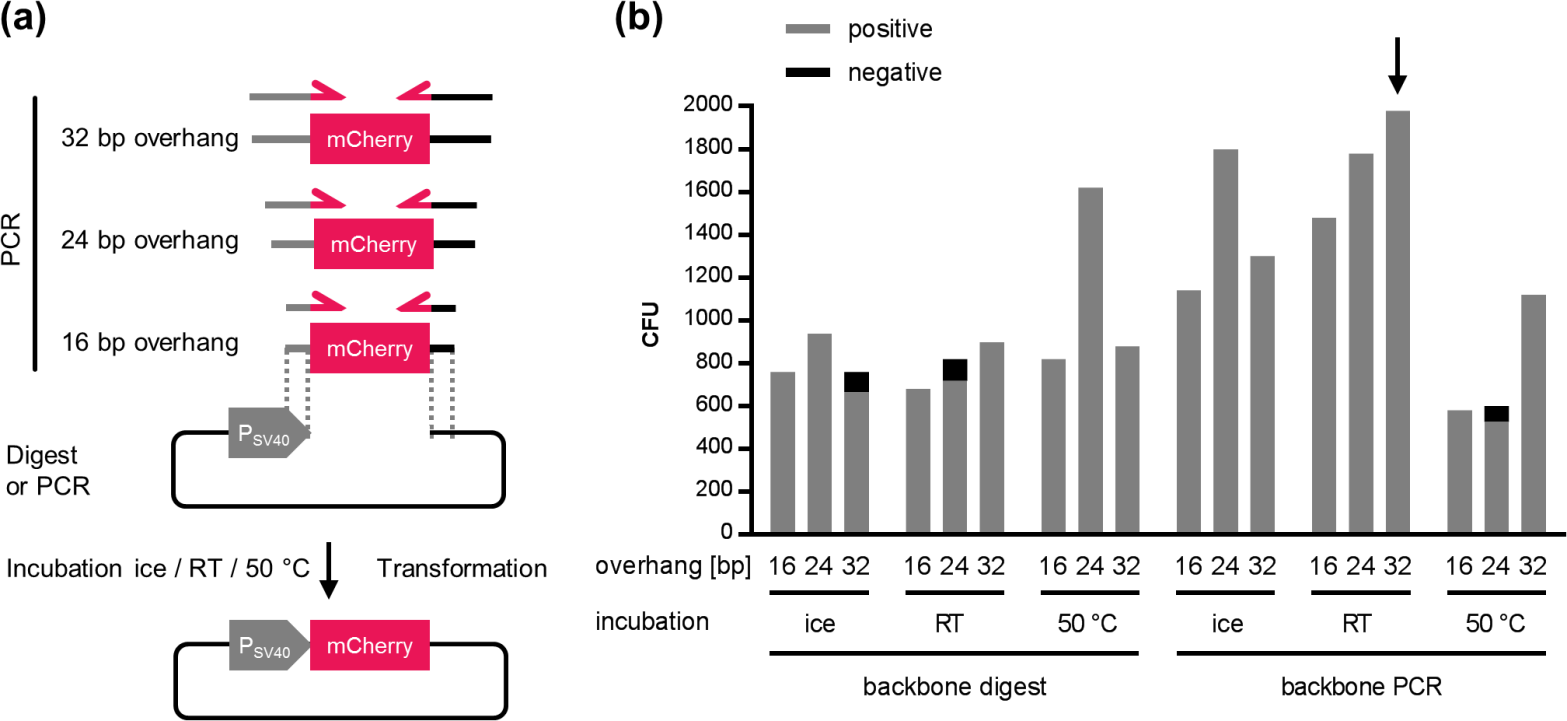
AQUA Cloning conditions. **(a)** The DNA fragment encoding the red fluorescent protein mCherry was PCR amplified with flanking extensions of 16, 24, or 32 bp of homologous sequence overlaps to an SV40 promoter-driven mammalian expression vector which was digested, or PCR amplified. AQUA Cloning was performed with pre-incubations on ice, at room temperature (RT), or at 50°C. DNA mixtures were transformed into chemically competent *E*. *coli* Top10 cells and colonies were obtained the next day. **(b)** Typical numbers of colony forming units (CFU) obtained from each condition. The highest number of CFU was derived from a pre-incubation at room temperature and with 32 bp of shared homology with PCR originating DNA fragments (arrow). The accuracies for each condition were determined by analytical colony PCR from eight selected clones and were extrapolated to the total number of CFU (grey). Abbreviations: P_SV40_, simian virus 40 early promoter.

Assembly cloning is increasingly replacing conventional cloning methods for reasons of convenience and performance. In order to test AQUA for cloning efficiency, we first performed the *de novo* assembly of a plasmid with two DNA fragments that were either prepared by PCR, or alternatively originated from an enzymatic digestion of prepared plasmids. The red fluorescent protein mCherry was chosen to be inserted into an SV40 promoter-driven mammalian expression vector (Fig 2A). For introducing regions of homology to a linearized expression vector, oligonucleotides were designed to anneal to the extremes of a gene of interest (GOI) to be cloned with 5’-extensions attached to the primer pairs. Gel-purified DNA fragments were mixed in water at a molar ratio of 3:1 (insert:vector) to prevent the limitation of insert fragments, thereby minimizing unspecific self-ligation of the antibiotic resistance-containing vector backbone. We hypothesized that the length of the homologous region shared by two adjacent DNA fragments, might correlate with the efficiency of correct plasmid assembly. Further, a proper premixing of all DNA fragments might contribute to the uptake of all required parts upon transformation into a single *E*. *coli* cell. A three-parameter screen was undertaken (i) varying the length of overlaps, 16 bp, 24 bp or 32 bp, (ii) the source of the DNA fragments (PCR or digest) and (iii) the temperature at which the DNA mixtures were incubated prior to transformation. The temperatures varied from incubation on ice up to 50°C, where partial DNA denaturation might favour a homologous sequence-determined association between the DNA molecules. DNA mixtures were transformed into an in-house prepared standard chemically competent *E*. *coli* cloning strain (TOP10). Colonies for each condition were obtained the next day and subsequently analyzed for correct assembly by analytical colony PCR. Interestingly, positive clones were obtained under all conditions tested ranging from 525 colony forming units (CFU) (PCR, 24 bp, 50°C) up to 1,980 CFU (PCR, 32 bp, room temperature [RT]) with highest accuracy for most conditions. This indicates a very robust behaviour to variations in the length of homologous sequence (Fig 2B). Accuracies were determined by analytical PCR from eight selected clones of each condition (Fig A in S1 File). For the optimal condition where most colonies were obtained (RT, 32 bp homology, PCR derived DNA) a clone was subsequently confirmed by comprehensive sequencing. Digested vector DNA yielded slightly fewer colonies in comparison to PCR-amplified plasmid backbones, denoting a higher tendency towards self-ligation, as determined from transformations of linearized backbone only (Fig 2B, Table B in S1 File). The highest number of colonies was obtained with a pre-incubation step at room temperature with 32 bp of shared overlapping sequence, and with DNA derived from PCR amplification. This condition was used in all further experiments. However, the PCR amplification of large vector sequences might introduce undesired mutations, some of which may influence the proper functionality of the genetic elements comprised. Depending on the application, a vector digestion might therefore be the preferred method of choice for vector preparation.

#### Screen for AQUA Cloning efficiency among common lab-strains of E. coli

In order to evaluate the broad utility of AQUA Cloning, different common commercially available strains of *E*. *coli* were tested.

Chemically competent cells of the strains TOP10, NEB5α, NEB10β, BL21 (DE3) and JM109 were used and the 2-DNA fragment cloning was carried out in parallel as previously described. Correct assembly was mediated by all strains tested, with efficiencies varying in terms of yield. TOP10 and NEB10β yielded the highest numbers of CFU (both > 1,000 CFU) while only as few as nine colonies were obtained from the expression strain BL21 (DE3). The accuracy of correct assembly, however, remained high among all strains (83–100%). The number of CFU obtained did not correlate well with the competency of each particular strain as given by the manufacturer, indicating a variation in the suitability for AQUA Cloning among the strains (see Table 2, Fig B in S1 File). From the results obtained in this screen, we conclude a wide applicability of AQUA cloning across most common *E*. *coli* lab strains, with NEB10β and TOP10 strains yielding the highest CFU numbers.

**Table 2:** Screen for AQUA Cloning efficiency among common lab-strains of *E*. *coli*. The cloning of the 2-DNA fragment assembly (see Fig 2) was performed at room temperature with 32 bp of homology and with DNA produced by PCR. Different commercially available chemically competent strains were tested along the TOP10 cells prepared in our lab. The efficiency was determined by analytical colony PCR of 12 clones (BL21 (DE3), 9 clones), see Fig B in S1 File.

Strain	N(CFU)	Competency (CFU/μg)	efficiency
TOP10 (self-prepared)	1160	0.6 x 10^7^	11/12
TOP10 (commercial)	233	> 10^9^	10/12
NEB5α (commercial)	283	1–3 x 10^9^	11/12
NEB10β (commercial)	1073	1–3 x 10^9^	12/12
BL21(DE3) (commercial)	9	1–5 x 10^7^	9/9
JM109 (commercial)	92	10^8^	12/12

#### De novo assembly of 4-DNA fragments

**Fig 3:**
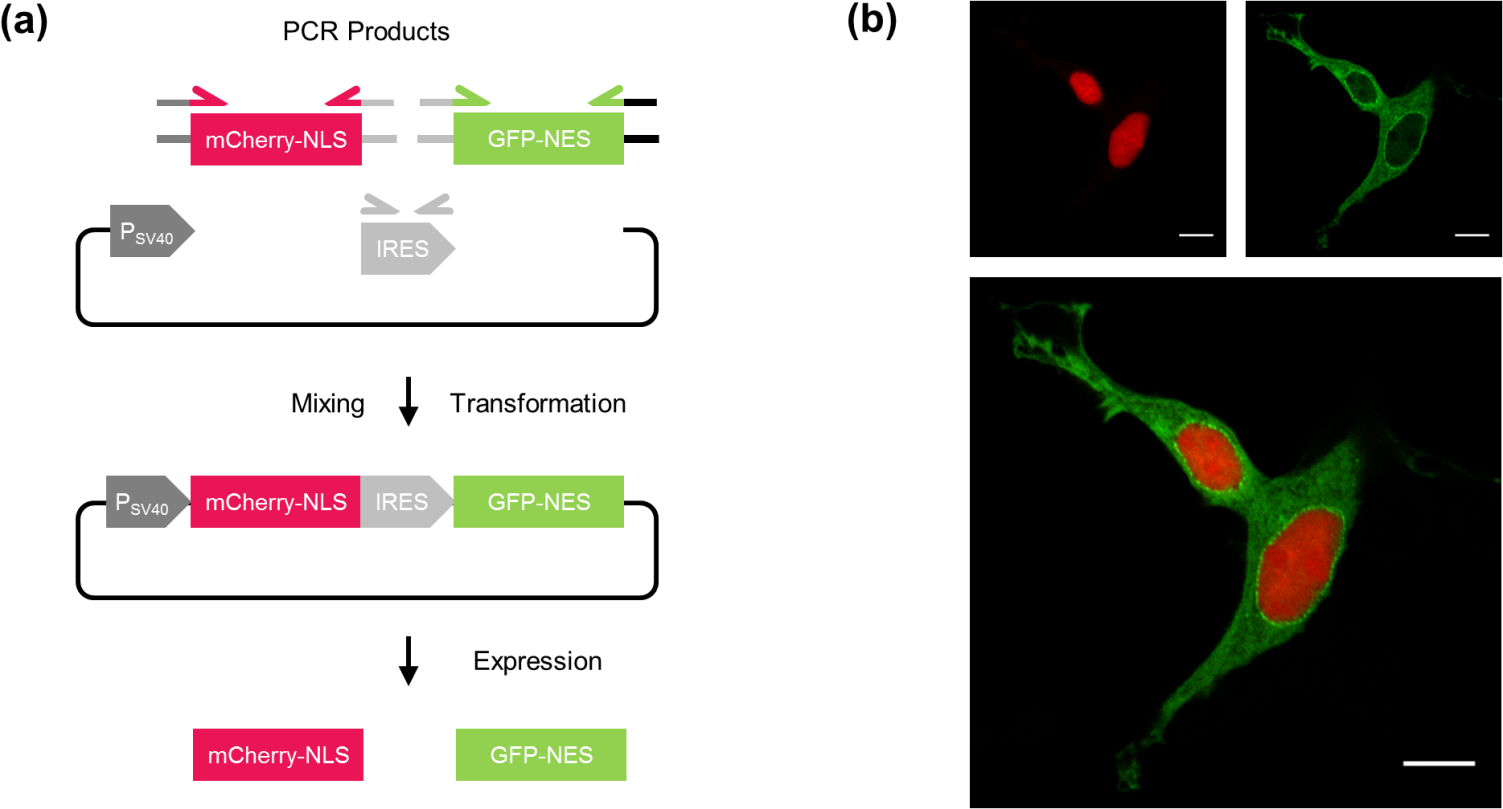
4-fragment assembly. **(a)** A quadruple *de novo* assembly was designed by generating four PCR amplified linear DNA fragments (i) mCherry with an NLS sequence, (ii) an IRES element for bicistronicity, (iii) EGFP with an NES sequence and (iv) the P_SV40_ promoter-driven mammalian expression vector. Each pair of adjacent fragments shared 32 bp of overlapping sequence. **(b)** Experimental verification of the correct assembly in (a) by transient transfection into HEK-293T cells followed by confocal imaging. Scale bar, 10 μm. Abbreviations: IRES, internal ribosomal entry site; NES, nuclear export signal; NLS, nuclear localization signal, P_SV40_, simian virus 40 early promoter.

To demonstrate the ability of AQUA Cloning to *de novo* assemble multiple fragments in a one-pot cloning step, a bicistronic mammalian expression vector was designed, comprising the red and green fluorescent proteins, mCherry and EGFP. For an expression control, we fused a nuclear localization signal (NLS, PKKKRKV) and nuclear export signal (NES, MTKKFGTLTI) to the C-terminal ends of mCherry and EGFP, respectively. Further, both genes were separated by an internal ribosome entry site (IRES) for bicistronicity. The circular expression plasmid (Fig 3A) is thus assembled by four DNA fragments. Upon correct assembly of the DNA fragments, mCherry is expected to be localized exclusively in the nucleus, and EGFP in the cytoplasm when transfected into mammalian cells.

Upon AQUA Cloning, 18 colonies out of 360 were screened for correct assembly by analytical PCR (Fig C in S1 File). An accuracy of 83% was determined, with 15 out of 18 colonies being positive. The functionality of a positive clone was confirmed experimentally, by transient transfection of the obtained plasmid into HEK-293T cells. The localization of the expressed proteins within the cells was recorded by confocal imaging (Fig 3B). The fluorescence images revealed a strong red and green fluorescence signal within the nucleus and cytoplasm, respectively. This followed the expectation for mCherry and EGFP protein localization and confirmed the correct assembly of the DNA fragments.

### Further applications of AQUA Cloning

#### Deletion and insertion of a sequence, site-directed mutagenesis

In addition to the assembly of different DNA fragments into a circular product, AQUA Cloning is also suitable for the circularization of a single PCR product with flanking homologous sequences. In this way, several modifications of the template plasmid can be undertaken and are demonstrated in this section. This includes the insertion and deletion of a sequence but also site-directed nucleotide substitutions.

Annealing of complementary oligonucleotides and subsequent ligation into digested plasmids is often the method of choice to insert short DNA sequences, such as tags or localization signals, into pre-existing plasmids. This approach, however, is strictly dependent on the presence of appropriate restriction sites.

**Fig 4:**
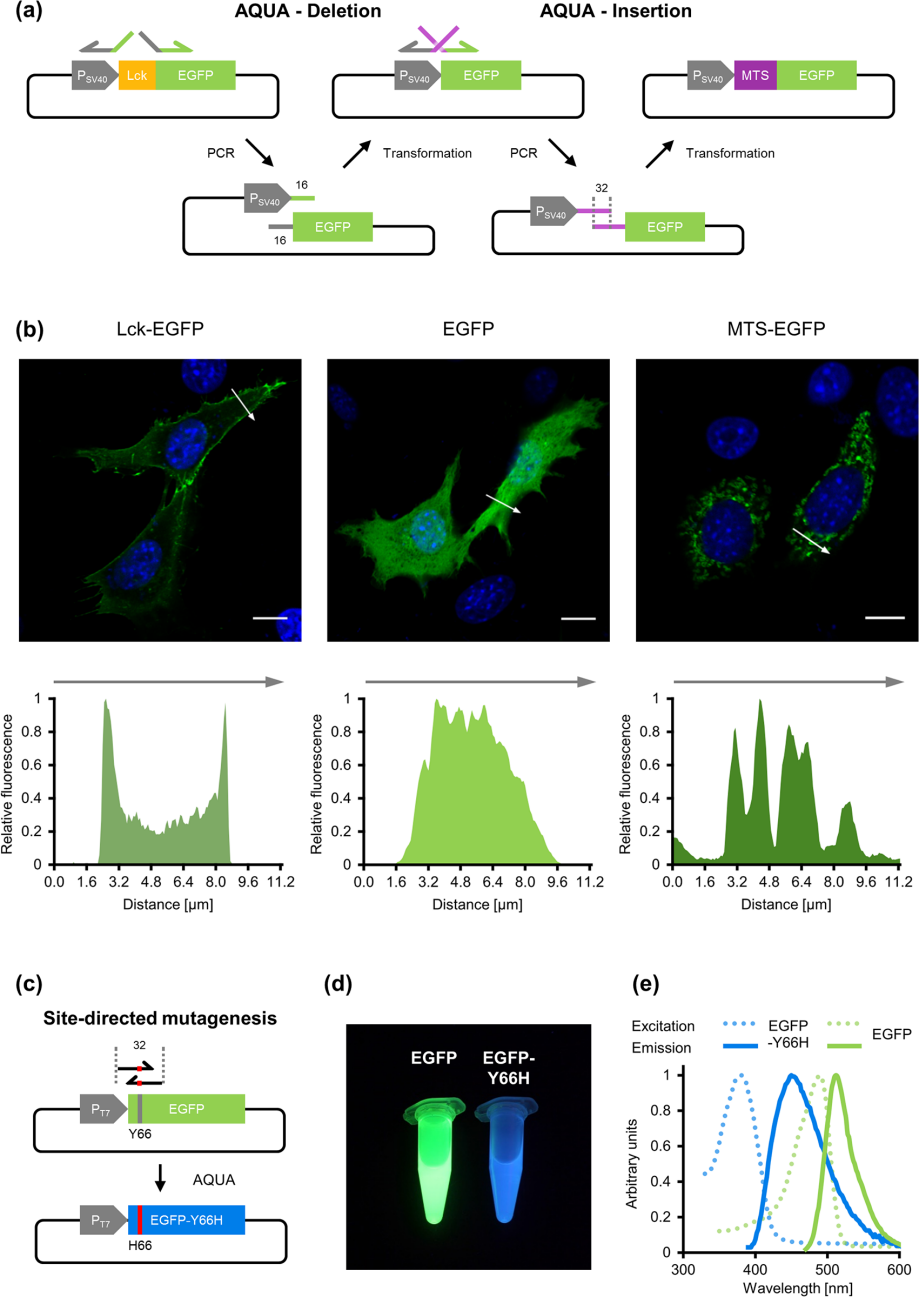
Site-directed mutagenesis with AQUA Cloning: deletion-, insertion- and point-mutagenesis. **(a)** For targeted deletion of the Lck membrane anchor from an Lck-fused EGFP, a primer pair was designed for whole-plasmid PCR with the forward primer annealing to EGFP and sharing overlaps to the P_SV40_ promoter region. The reverse primer anneals to the P_SV40_ promoter and shares overlaps to EGFP. The resulting PCR product hence excludes the Lck membrane anchor and the extremes are complementary by 32 bp allowing AQUA assembly into a circular plasmid for cytosolic expression of EGFP. The resulting EGFP expression plasmid was further used as a PCR template with a primer pair where the 5’-extensions were replaced with a mitochondrial target signal (MTS) which localized expressed EGFP into the mitochondria of the host cell. The flanking MTS sequence of the resulting PCR product serves as region of homology for AQUA Cloning to construct the expression plasmid with the MTS sequence inserted. **(b)** Confocal imaging after transient transfection into NIH/3T3 cells reveals successful AQUA Cloning: the deletion of the Lck-membrane anchor resulted in cytosolic expression of EGFP (middle) of previously membrane localized protein (left), and the insertion of a MTS in recruitment of EGFP into the mitochondria (right). Scale bars, 10 μm. Corresponding fluorescence intensity profiles are given below each figure. **(c)** For site-directed substitution of a single amino acid in EGFP, a primer pair was designed surrounding the target site. 5’-extensions were attached to encode the Y66H substitution (leading to blue fluorescence) and at the same time contributing a region of homology for AQUA Cloning of a single linear PCR product. **(d)** Green and blue fluorescence of recombinant purified EGFP and EGFP-Y66H, respectively upon excitation with 366 nm light. **(e)** Excitation and emission spectra of EGFP and EGFP-Y66H.

AQUA Cloning allows for an alternative way of insertion, and also the deletion, of oligonucleotide-encoded genetic information: PCR amplification of the whole plasmid leads to a flanking of both ends of the resulting product with the sequence to be inserted. In this way, a linear PCR product is obtained that shares complementary sequence for AQUA circularization at the extremes (see Fig 4A).

Moreover, the deletion of any sequence of interest may be facilitated with a single PCR reaction (Fig 4A). As a proof of concept, an N-terminal Lck-derived membrane anchor (MGCWCSSNPEDD), fused to EGFP, was removed by adjacent oligonucleotide positioning (Fig 4A, left). AQUA Cloning of the obtained PCR product resulted in a plasmid encoding EGFP exclusively present in the cytosol, as visualized by confocal microscopy (Fig 4B, left and middle). The resulting plasmid was next used as a PCR template to demonstrate the insertion of a sequence of interest into the vector. The linear PCR product was flanked by a mitochondrial target signal (MTS, MSVLTPLLLRGLTGSARRLPVPRAKIHSL), including 32 bp of homologous sequence for AQUA Cloning. Consequently, microscopic analysis confirmed mitochondrial localized EGFP (Fig 4B, right).

Substitutions of single amino acids are often aimed to confer targeted alterations to proteins such as loss-of-function, or gain-of-function mutations. Commercial site-directed mutagenesis kits are offered to introduce specific point mutations by PCR [20].

AQUA Cloning represents an inexpensive and efficient alternative for performing site-directed substitutions. Introducing single base substitutions into the oligonucleotide-provided homologous regions, followed by whole-plasmid amplification and AQUA assembly, is a fast way to obtain desired mutants. The *in vivo* recombination-based mechanism of AQUA Cloning for mutagenesis aligns well with recent evidence revealing the actual molecular principle underlying the QuickChange mutagenesis protocol and its improvements which was misinterpreted for years [21]. We have now expanded this principle for meaningful cloning applications as laid out in this article. As a proof of principle, a bacterial expression plasmid encoding EGFP was used as a PCR template to introduce the Y66H mutation, leading to a protein with blue-shifted fluorescence [22] (Fig 4C). In order to keep the AQUA protocol entirely enzyme-free and due to the exponential amplification of the PCR product combined with gel purification, DpnI digestion of the parental plasmid was omitted. However, additional DpnI treatment might be advantageous in case too much template plasmid is transferred giving rise to false positive colonies. As colony screening by PCR or restriction digest may be challenging when the product only differs marginally from its original plasmid, two clones were stochastically selected and sequenced. A positive clone was used for protein expression in *E*. *coli* and the mutated protein was purified by affinity chromatography to compare its fluorescence properties with the wild-type (Fig 4D and Fig 4E).

Taken together, AQUA Cloning provides the simplest means of realizing sophisticated mutagenesis that exceeds the potentials of other existing mutagenesis protocols in terms of flexibility, costs and applicability.

#### AQUA Expression—combined cloning and expression

DNA cloning is often considered a time-consuming process that precedes the actual experiments. In some cases, as for the production of recombinant protein in *E*. *coli*, the time required for cloning may even exceed the time required for the experiment in which the recombinant protein may be used for.

**Fig 5:**
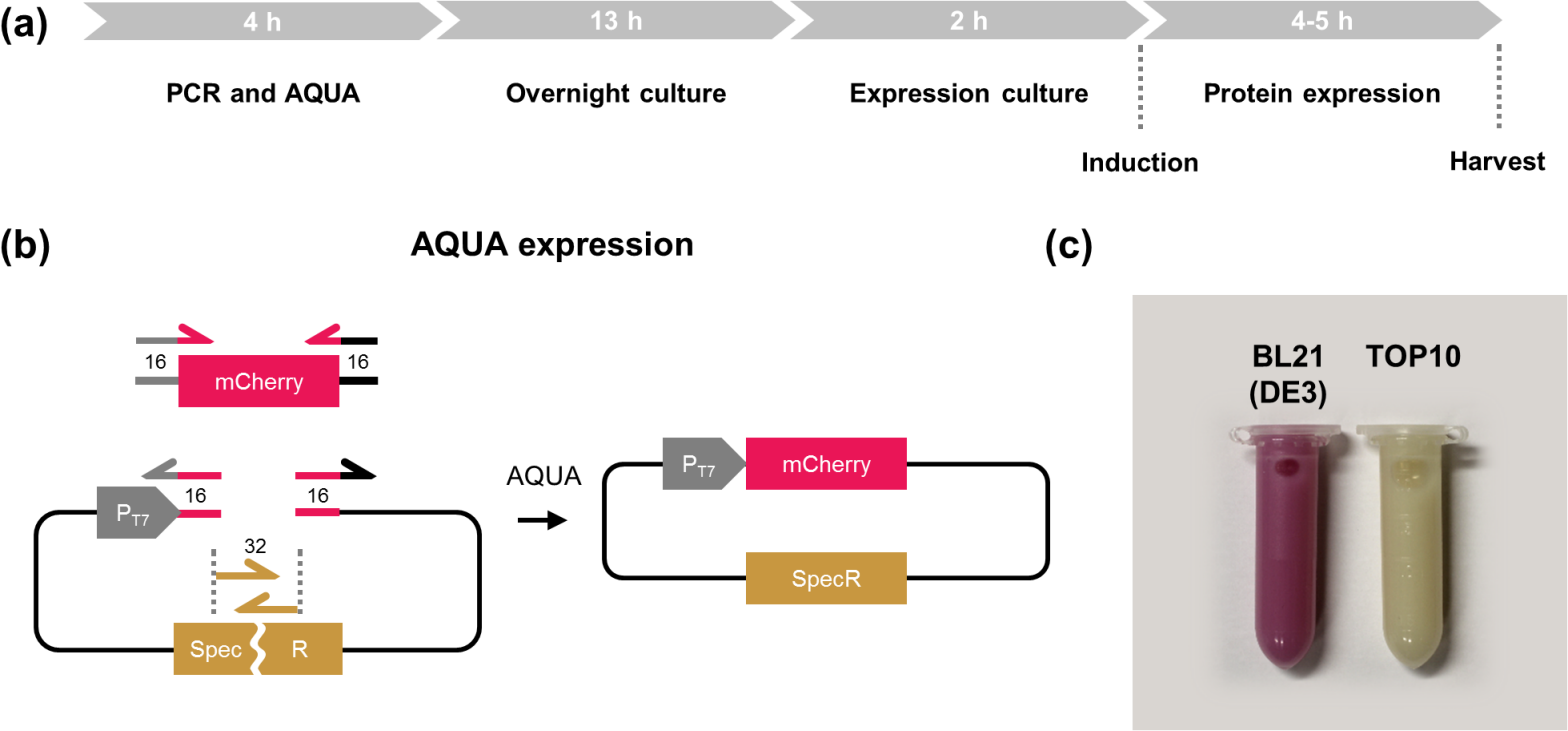
AQUA Expression—combined cloning and protein expression. **(a)** Timeline for AQUA Expression. Cloning and production of recombinant protein in *E*. *coli* may be performed within 24 h starting with the PCR until the bacteria are harvested the next day. **(b)** A 3-DNA fragment cloning was performed by inserting the coding sequence for the red fluorescent protein mCherry into a bacterial T7 promoter-driven expression vector. The vector was split into two parts within the resistance gene for the antibiotic spectinomycin. Therefore, only correctly assembled fragments allow cell growth. **(c)** AQUA Expression in the expression strain BL21 (DE3) results in red colored bacteria due to mCherry protein production, while the TOP10 strain—lacking the required T7 RNA polymerase—remains colorless.

With AQUA Cloning robustly assembling circular plasmids *in vivo* within the most common strains of *E*. *coli*, we aimed to reduce the labor and time required for the production of recombinant protein to an absolute minimum. By transferring the DNA assembly reaction into the expression host, it becomes possible to combine cloning and expression in a single cell, thus performing cloning and expression of recombinant protein within 24 h, from one day to the next (see Fig 5A).

To demonstrate ‘AQUA Expression’, a plasmid consisting of three DNA fragments was assembled by AQUA Cloning in the expression strain BL21 (DE3). One fragment contained the coding sequence for the red fluorescent protein mCherry, while the two remaining DNA segments comprised a bacterial expression vector split within the resistance gene for the antibiotic, spectinomycin (Fig 5B). With this approach, the method entails an intrinsic positive selection for *in vivo* assembly, as only clones performing the re-assembly of the resistance gene will emerge in the presence of the antibiotic. Upon AQUA Cloning, the transformed cells were cultivated overnight in LB-medium containing the antibiotic and were then used the next morning to inoculate an expression culture. Expression was induced at an appropriate optical density and harvested in the afternoon. The red color of the harvested bacteria resulting from mCherry expression clearly indicated that correct assembly had occurred. As expected, a control strain (TOP10) treated identically did not turn red after induction, as these cells lack the T7 RNA polymerase required for expression (Fig 5C).

#### Combinatorial AQUA Cloning for plant cell systems

The experimental characterization of protein variants in parallel within a particular experimental system requires each of the variants to be cloned in advance. As experimental complexity increases, this may result in a combinatorial muddle, slowing down efficient progress. This is for instance the case in metabolic engineering approaches where different versions of enzymes must be cloned and screened for maximization of the production chain yield [23]. To overcome the experimental limitation given by the cloning process, an adequate assembly method for the simplified generation of genetic combinatorial libraries must be implemented.

AQUA Cloning relies on short homologous DNA sequences to guide the order of fragment assembly. Libraries of variants of a particular gene may be created by PCR, employing the same pair of oligonucleotides that will share identical extremes for AQUA Cloning, thus providing a convenient way of combinatorial variant generation within complex plasmid constructs.

**Fig 6:**
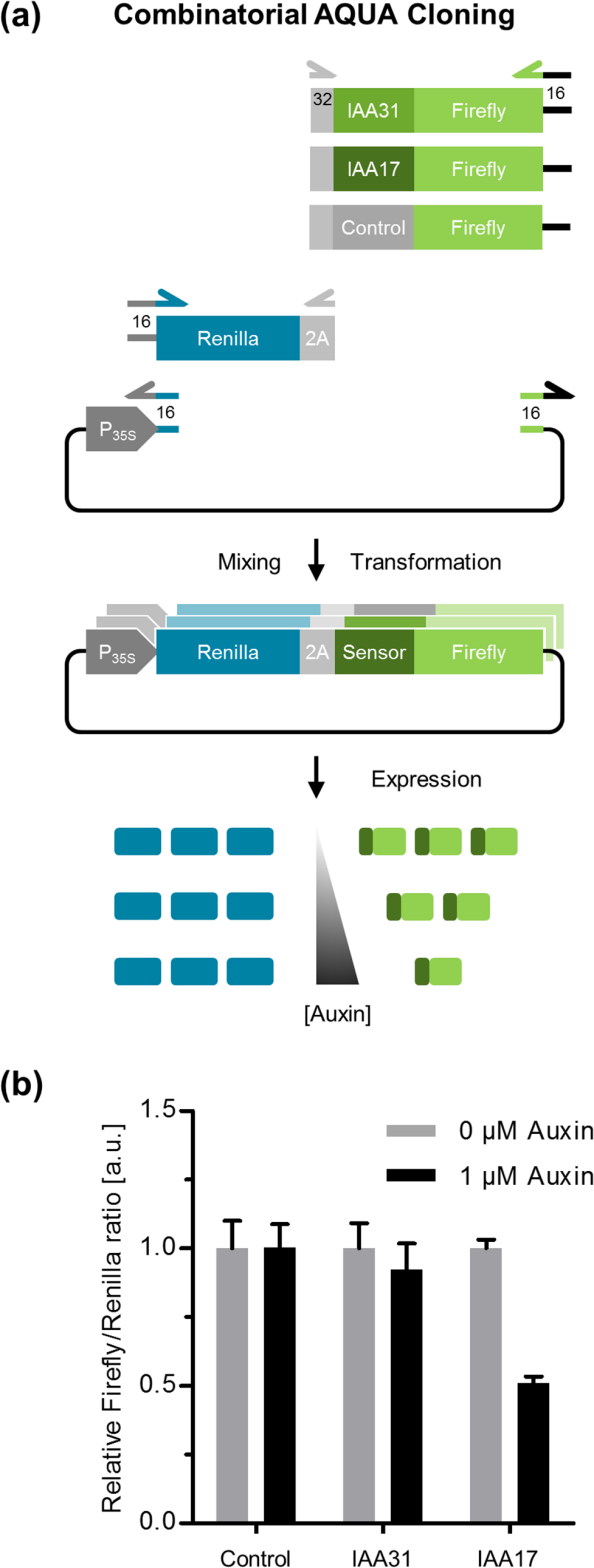
Combinatorial AQUA Cloning for plant cells. (a) For a combinatorial cloning of 3 plasmids, each originating from 3 linear DNA parts (variants of a ratiometric auxin sensor) out of a pool of 5 parts, were constructed. The consensus sequences derived from the Aux/IAA 31 and Aux/IAA 17 proteins, or an auxin insensitive sequence, each fused to the firefly luciferase were prepared. The fragments were cloned together with the renilla luciferase, separated by a 2A peptide for equimolar expression, into a 35S promoter-driven plant expression vector. Expression of the sensor constructs in plant cells should enable the auxin-dependent degradation of firefly, while renilla remains unaffected for intrinsic signal normalization. **(b)** Relative firefly:renilla ratios obtained from protoplasts expressing the sensor variants after 1 h of auxin treatment. Error bars represent standard error of the mean, n = 6.

To demonstrate the applicability of AQUA Cloning for parallel, combinatorial cloning, we generated alternative variants of a plant hormone sensor for the quantitative detection of auxin in plant cells. To this end, we resorted to a genetically encoded ratiometric auxin sensor based on plant auxin sensing molecular mechanisms [18]. A co-receptor complex involving Aux/IAA family proteins is responsible for auxin perception by plant cells. In the presence of auxin, Aux/IAAs are ubiquitylated and targeted for proteolytic degradation by the 26S proteasome. The sensor consists of a sensing module, based on a 13 amino acid consensus sequence of Aux/IAA proteins sufficient for auxin binding, fused to firefly luciferase (sensor) [18]. Upon increasing concentrations, auxin targets the Aux/IAA-based sensor for proteasomal degradation leading to a decreased firefly luminescent signal (readout). In order to obtain a quantitative determination, the construct further encodes a renilla luciferase as a normalising element. Renilla is separated from the sensor element by a picornaviral 2A peptide that mediates co-translational proteolytic cleavage of both polypetides leading to equimolar expression of the sensor module fused to the firefly luciferase that is then degraded in the presence of auxin. The renilla luciferase is not degraded and is hence normalising the readout signal (Fig 6A). Arabidopsis encodes 29 different Aux/IAA variants spanning a wide spectrum of binding affinities with Kd values ranging from 10 nM up to 1,000 nM [24]. By using sensor modules (13 amino acids consensus sequences) from different Aux/IAA proteins, sensors of various sensitivities could be engineered. AQUA Cloning was utilized as a helpful and simple cloning method for generating a combinatorial library of auxin sensors.

Firstly, a library containing two variants of Aux/IAA consensus sequences, IAA31 and IAA17 with K*d* values for auxin binding of 33 nM and > 1000 nM, respectively, and a control sequence (Control, auxin insensitive) containing seven pairs of glycines and alanines was created by PCR as fusions to the firefly luciferase. The different variants present in the library were then inserted into a 35S promoter-driven plant expression vector and the renilla luciferase was separated by the 2A peptide coding sequence (Fig 6A). This resulted in three versions of the plant auxin sensor. The control sensor is insensitive to auxin and should exhibit a constant ratio of firefly:renilla bioluminescence, irrespectively of auxin administration. The IAA31 sensor that is based on the Aux/IAA31 consensus sequence exhibiting a high K*d* value for auxin binding, should only marginally respond to auxin compared to the IAA17 sensor with a much lower K*d* value.

Upon transient transformation of the three sensor constructs into protoplasts of *A*. *thaliana*, indeed, the sensors behaved as expected in their response towards the addition of 1 μM auxin to the medium for 1 h. Protoplasts that were kept untreated were used as a reference in this experimental setup (Fig 6B). This demonstrates in a proof-of-principle application the suitability of AQUA Cloning for the easy combinatorial cloning of three plasmids, each originating from three linear DNA segments out of a pool of five DNA fragments.

#### Multi-fragment assembly for the development of a construct for light- and chemically-regulated logic operations in mammalian cells

The rational *de novo* design and development of genetic circuits that sense environmental stimuli and respond logically, with a well-defined output, is a key field of application in synthetic biology [25]. These computational networks are highly modular and allow the combinatorial design of complex computations from unique genetic logic gates. Examples include switches [26], memory devices [27] and oscillators [28] that together enable the implementation of programmable functionality into living cells [29]. The underlying molecular design is complex in terms of the number and the modularity of the genetic components involved, particularly cumbersome when relying on standard cloning techniques. Avoiding the construction of intermediate plasmids and reducing the time and workload of such complex cloning approaches to a one pot, one step seamless, enzyme-free assembly is one of the crucial advantages of AQUA Cloning.

**Fig 7:**
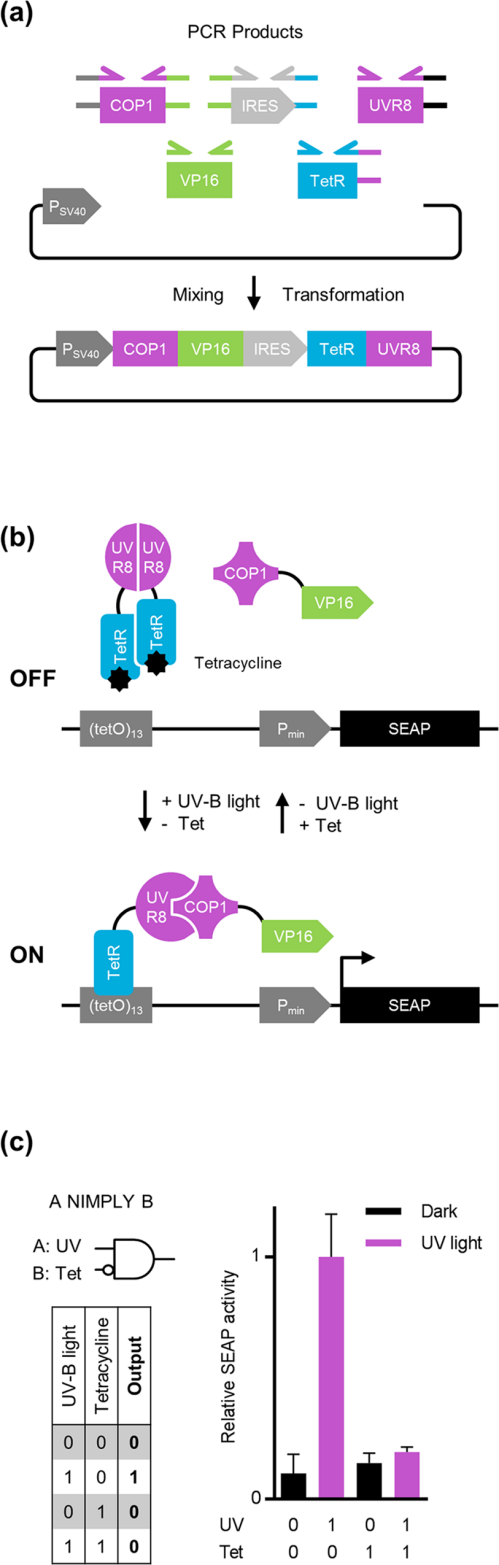
Multi-fragment assembly for the development of a light- and chemically-regulated Boolean logic operation. **(a)** Each DNA fragment for the assembly was PCR-amplified with 32 bp of homology region at each fusion-site as indicated schematically. The first cistron is composed of a fusion of the tetracycline repressor protein TetR to the UVR8 ultraviolet B (UV-B) light photoreceptor. The second cistron, separated by an IRES element, comprises a fusion of the interaction factor COP1 to the transcriptional activation domain VP16. AQUA Cloning of the 6 DNA fragments yields the desired bicistronic mammalian expression vector. **(b)** Functional principle of the light- and chemically-regulated A NIMPLY B logic gate. In the absence of tetracycline, TetR binds to its cognate operator element (TRE) of an appropriate reporter plasmid. The UV-B light mediated activation of UVR8 provokes the dissociation of the otherwise homodimeric conformation and allows the interaction and hence the recruitment of COP1 which is fused to the VP16 activation domain. The close proximity of the transactivator VP16 to the minimal promoter (P_min_) mediates the transcription of the encoded SEAP reporter gene under UV-B light irradiation and without the addition of tetracycline. **(c)** Truth table (left) and experimental results (right) of the A NIMPLY B gate. SEAP activity is detected only in the presence of UV-B light and in the absence of tetracycline, validating a functional A NIMPLY B logic gate. Abbreviations: COP1, CONSTITUTIVELY PHOTOMORPHOGENIC 1; IRES, internal ribosome entry site; P_min_, minimal human cytomegalovirus immediate early promoter; P_SV40_, simian virus 40 early promoter; SEAP, human placental secreted alkaline phosphatase; TetR, tetracycline repressor protein; UVR8, UV resistance locus 8 protein; VP16, viral transactivation domain.

To demonstrate the applicability of AQUA Cloning for the design of a complex Boolean logic gate, we developed a bicistronic plasmid that encodes a light- and chemically-regulated A NIMPLY B gate, the output of which is only true when A is true and B is false. To this end, we harnessed the previously published ultraviolet-B (UV-B)-responsive gene expression system. This is based on the UV-B light-dependent interaction of the UV-B light photoreceptor protein, UV resistance locus 8 (UVR8), with CONSTITUTIVELY PHOTOMORPHOGENIC 1 (COP1) from *Arabidopsis thaliana* [17]. The UVR8 receptor was fused to the tetracycline repressor protein TetR in order to mediate both binding of UVR8 to a genetic tetracycline response element (TRE) and sensitivity towards tetracycline. COP1 on the other hand was fused to the transcriptional activation domain VP16 from *Herpes simplex*. Both cistrons were combined in a single mammalian expression vector mediated by an IRES element, resulting in a 6-fragment *de novo* assembly (Fig 7A). UV-B light exposure switches gene expression on, but the addition of tetracycline to the cell-culture medium prevents any binding of the activation complex to the TRE of a cognate reporter plasmid and hence, acts dominant over UV-B light activation (Fig 7B).

Upon transient transfection of CHO-K1 hamster ovary cells with the UV-B system, only UV-B light illuminated cells that were not treated with tetracycline expressed the human placental secreted alkaline phosphatase (SEAP) reporter. CHO-K1 cells that were not illuminated with UV-B light showed only basal expression levels. This indicated that the expression vector containing the A NIMPLY B logic components was successfully assembled with AQUA (Fig 7C). Taken together we demonstrate how complex genetic networks encoded in a single plasmid may be constructed employing the simplicity and convenience of AQUA Cloning. Not being restricted by the indispensable singularity of restriction sites and taking advantage of its ease and low-priced multi-DNA fragment assembly, AQUA Cloning is a versatile, simple and powerful method for everyday cloning.

## Discussion

Assembly cloning is increasingly replacing conventional restriction enzyme and DNA ligase-mediated cloning. The reasons for this are the generally higher efficiency of correct assemblies, the seamless in-frame fusion of multiple genetic elements within a single step, the dispensability of (unique) restriction enzyme sites and the reduced efforts in terms of labor along with an overall economization and an optimized performance.

AQUA Cloning, described in this work, overcomes some of the drawbacks of current assembly cloning methods. It allows the fast, enzyme free assembly of several linear DNA molecules with short flanking homologous regions into circular plasmids.

There are alternative enzyme-free cloning protocols that have successfully simplified conventional cloning strategies, however they require additional experimental steps, including extra PCR products and denaturation/re-annealing steps for heteroduplex formation [10–12]. This work follows up and expands recent reports of *E*. *coli in vivo* cloning [14]: in addition to facilitating simple cloning (*e*.*g*. 2–3 fragments), we demonstrate here the suitability and versatility of *in vivo* recombination-based cloning for the simple, reliable and time/cost-efficient realization of more complex tasks. This includes and is not limited to a wide range of applications ranging from (i) the convenient modification of existing plasmids (insertions, deletions, or substitutions of DNA sequences), (ii) the one step cloning and expression of proteins, up to (iii) the *de novo* combinatorial design and assembly of multiple-fragment constructs comprising at least 6 DNA parts. Therefore we anticipate a rising popularity of this method over existing enzyme-dependent and-independent protocols. We have investigated the efficiency and applicability, and found AQUA Cloning to be both simple and reliable. With virtually no preparation to be made in advance and the compatibility of standard chemically competent *E*. *coli* cells, AQUA Cloning exhibits advantages over other cloning methods. AQUA Cloning is independent of any restriction enzymes, DNA ligases, exonucleases or bacterial lysates that are required in other assembly cloning protocols [3–9,30–33]. The method is compatible with both linear DNA prepared from an enzymatic digestion or from amplification via PCR, facilitating the design phase. AQUA Cloning exhibits a low tendency towards self-assembly of vector fragments of 1.06% (optimized conditions: PCR, RT, 32 bp–Table B in S1 File) despite the omission in this study of previous dephosphorylation, underlining the simplicity of the workflow. AQUA Cloning is also compatible with the guidelines of various other cloning methods such as Gibson assembly, and hence, helpful design tools or existing DNA libraries for combinatorial assemblies can be well combined [23,34].

AQUA cloning relies on intrinsic processing mediated by *E*. *coli* upon transformation of linear DNA fragments. We have optimized the cloning efficiency and found it to be highest when employing 32 bp of shared homology. However, we also demonstrated that AQUA tolerates a range of homology from 16 to 32 bp in length and likely above (not tested), allowing an additional degree of flexibility. Second, with the simplicity given in the work-flow, AQUA Cloning remains highly versatile and may be used for the fast generation of complex cloning approaches involving insertion-, deletion- and point-mutagenesis as demonstrated with some examples reflecting typical needs in modern molecular and synthetic biology. On the other hand, even complex multi-fragment cloning—here tested up to six DNA parts—can be performed. This is suited *e*.*g*. for combinatorial approaches for metabolic engineering or the development of computational networks. This was demonstrated with a light- and chemically-controlled switch for the development of an A NIMPLY B logic gate encoded in a single plasmid and the combinatorial assembly of three variants of a quantitative auxin sensor for plant cells. The method shows a broad compatibility with most widely used lab-strains of *E*. *coli*, including TOP10, NEB5α, NEB10β, BL21 (DE3) and JM109. AQUA Cloning using the standard expression strain BL21 (DE3) reduces the time required for cloning virtually to zero, as a combined cloning and expression of recombinant proteins may be performed within 24 h.

Except for simple subcloning where DNA oligonucleotide synthesis and PCR amplification is not required, we anticipate the rising popularity in assembly cloning methods that will rapidly replace conventional cloning. Any standard molecular biology laboratory is readily equipped for AQUA Cloning and, within the repertoire of assembly cloning protocols, it is a straightforward alternative and compatible approach next to commercial methods. AQUA Cloning excels in terms of wide application scope, simplicity and its cost efficiency.

## Supporting Information

S1 File Supporting Information. Plasmids and oligonucleotides used in this study **(Table A)**. AQUA Cloning conditions of PCR derived DNA fragments **(Table B)**. Analytical colony PCR for optimized AQUA Cloning conditions **(Fig A)**. Efficiency of the 2-fragment assembly in different common strains of *E*. *coli* **(Fig B)**. Efficiency of the 4-fragment de novo assembly **(Fig C)**.
(DOC)
