# Supplementary material for: AQUA Cloning: A Versatile and Simple Enzyme-Free Cloning Approach
Source: PLoS One. 2015 Sep 11;10(9):e0137652. doi: 10.1371/journal.pone.0137652 (PMC4567319; doi:10.1371/journal.pone.0137652)
Supplement: S1 File — Plasmids and oligonucleotides used in this study (Table A). AQUA Cloning conditions of PCR derived DNA fragments (Table B). Analytical colony PCR for optimized AQUA Cloning conditions (Fig A). Efficiency of the 2-fragment assembly in different common strains of E. coli (Fig B). Efficiency of the 4-fragment de novo assembly (Fig C). (DOC) [file pone.0137652.s001.doc]

**AQUA Cloning: a versatile and simple enzyme-free cloning approach**

**Hannes M. Beyer^1,2^, Patrick Gonschorek^1^, Sophia L. Samodelov^1,2^, Matthias Meier^3,4^, Wilfried Weber^1,2,3^ and Matias D. Zurbriggen^1,3^***

^1^ Faculty of Biology, University of Freiburg, Freiburg, Germany

^2^ Spemann Graduate School of Biology and Medicine (SGBM), University of Freiburg, Freiburg, Germany

^3^ BIOSS Centre for Biological Signalling Studies, University of Freiburg, Freiburg, Germany

^4^ IMTEK, Department of Microsystems Engineering, University of Freiburg, Freiburg, Germany

* Corresponding author

E-mail: matias.zurbriggen@biologie.uni-freiburg.de (MDZ)

**Supplementary Information**

| **Supplementary Table A** | Plasmids and oligonucleotides used in this study. |
| --- | --- |
| **Supplementary Table B** | AQUA Cloning conditions. |
| **Supplementary Fig A** | Analytical colony PCR for optimized AQUA Cloning conditions. |
| **Supplementary Fig B** | Analytical colony PCR for screen of *E. coli* strains. |
| **Supplementary Fig C** | Efficiency of the 4-fragment de novo assembly. |

**Supplementary Table A. Plasmids and oligonucleotides used in this study.** If not indicated otherwise, all plasmids were cloned using AQUA Cloning.

| Plasmid / Oligo | Description | Ref. or source |
| --- | --- | --- |
| oHB304 | AGGTGATGTCCAACTTGATGTTGACG | This work |
| oMZ558 | CTCCGCCCCATGGCTGACTAATTTT | This work |
| pBS 35S: EGFP+ term | 35S promoter-driven plant expression vector for EGFP. | Yan *et al.*[1] |
| pCDF-Duet^TM^-1 | T7 promoter-driven empty bacterial expression vector. | Novagen® |
| pHB004 | **P_T7_::Avitag-TEV_CS_-mEGP-PIF6(N100)**  Bacterial expression vector encoding the hexahistidine-tagged monomeric green fluorescent protein mEGFP.  mEGFP was amplified from pMZ725 using the oligonucleotides oHB006 (5’- CGAAGGCGGCAG-CGCGGGCAGCGGTAGGAGCGGCGAAAATCTTTATTTTCAAGGTAGCGGCGGTAGCGGCG-CGGAAAGCGGTGGCATGGTGAGCAAGGGCGAGGAGC-3’) and oHB008 (5’-AGCAGAACCT-GCGGAGCCCTTGTACAGCTCGTCCATGCCGAG-3’). Additional sequence was attached using oHB007 (5’-GAAATAATTTTGTTTAACTTTAAGAAGGAGATATACATATGTCCGGCCTGAACGA-CATCTTCGAGGCTCAGAAAATCGAATGGCACGAAGGCGGCAGCGCG-G-3’) and oHB008. PIF6(N100) was amplified from pMH023 using oHB009 (5’-ATGGACGAGCTGTACAAGGGCTC-CGCAGGTTCTGCTGGT-3’) and oHB011 (5’-CTTCCTTTCGGGCTTTGTTAGCAGCCGGATCA-AGCTTTTAATGGTGATGGTGATGATGGTCA-ACATGTTTATTG-3’). The products were Gibson-cloned into H*ind*III/*Nde*I digested pWW301. | This work |
| pHB009 | Mammalian expression vector encoding the monomeric green fluorescent protein mEGFP fused to a nuclear export sequence (NES, MTKKFGTLTI, amino acid sequence) which was originally PCR amplified from pMZ725. | unpublished |
| pHB042 | P_SV40_-driven mammalian expression vector derived from *Xba*I/*Not*I digested pMZ333 with a C-terminal leucine zipper (Zip+) preceding a *Bam*HI restriction site. | unpublished |
| pHB080 | **P_SV40_::Lck-mEGFP-Zip(+)-pA**  SV40 promoter-driven mammalian expression vector encoding membrane-anchored monomeric EGFP with a carboxy-terminal leucine-zipper element (Zip+).  mEGFP was amplified from pMZ725 using the oligonucleotides oHB182 (5’-GGATCGAATTGCG-GCCGCAGGAGGCGCCACCATGGGCTGCTGGTGCAGCAGCAACCCCGAGGACGACGGTAGTGCTGGTAGTGCTGGTAGTGCTGGTATGGTGAGCAAGGGCGAGGAGC -3’) and oHB183 (5’-GATCCAGATCCGGATCCCTTGTACAGCTCGTCCATGCCGAG-3’) thereby attaching the Lck-derived myristoylation/palmitoylation sequence (MGCWCSSNPEDD, amino acid sequence). The product was ligated into *BamH*I/*Not*I digested pHB042. | This work |
| pHB351 | **P_SV40_::mCherry‑pA**  P_SV40_-driven mammalian expression vector encoding the red fluorescent protein mCherry.  mCherry was PCR amplified from pMZ701 using the oligonucleotides oHB305F (5’‑TCCCGGAT-CGAATTGCGGCCGCAGGAGGCGCCACCATGGTGAGCAAGGGCGAGGA‑3’) and oHB305R (5’‑GGGCTGCAGGTCGACTCTAGACTACTTGTACAGCTCGTCCATGCC‑3’) for creating a 16 bp overhang. To generate 24 bp of overhang, the oligonucleotides oHB306F (5’‑ATTTCAGG-TCCCGGATCGAATTGCGGCCGCAGGAGGCGCCACCATGGTGAGCAAGGGCGAGGA‑3’) and oHB306R (5’‑CGAAGCTTGGGCTGCAGGTCGACTCTAGACTACTTGTACAGCTCGTCCATGC-C‑3’) were used. To generate 32 bp overhangs, oHB307F (5’‑TGTCTTTTATTTCAGGTCCCGG-ATCGAATTGCGGCCGCAGGAGGCGCCACCATGGTGAGCAAGGGCGAGGA‑3’) and oHB307R (5’‑GTCTGGATCGAAGCTTGGGCTGCAGGTCGACTCTAGACTACTTGTACAGCTC-GTCCATGCC‑3’) were used. The backbone pMZ333 was either amplified using the oligo-nucleotides oHB308F (5’‑AGTCGACCTGCAGCCCAAGC‑3’) and oHB308R (5’‑GCAATTCGAT-CCGGGACCTG-AAAT-3’) or was digested with *Xba*I/*Not*I. | This work |
| pHB353 | **P_SV40_::mCherry‑NLS‑IRES‑mGFP‑NES‑pA**  Bicistronic SV40 promoter-driven mammalian expression vector encoding the red fluorescent protein mCherry fused to an nuclear localization sequence (NLS, PKKKRKV, amino acid sequence) and the monomeric green fluorescent protein mEGFP fused to an nuclear export sequence (NES, MTKKFGTLTI, amino acid sequence) separated by an internal ribosomal entry site.  mCherry-NLS was PCR amplified using the oligonucleotides oHB314 (5’‑TTTTATT-TCAGGTCCCGGATCGAATTGCGGCCGCAGGAGGCGCCACCATGGTGAGCAAGGGCGAGG-A‑3’) and oHB315 (5’‑GTTTTAAGCTTGGGCTGCAGGTCGACTCTAGACTACACCTTCCGCT-TTTTCTTGGGC‑3’) from pSJ027. The IRES sequence was PCR amplified from pKM022 using the oligonucleotides oHB310 (5’‑TCTAGAGTCGACCTGCAGCCC‑3’) and oHB311 (5’‑ATGAA-TTCGCGGCCGCAATCC‑3’). mEGFP-NES was PCR amplified from pHB009 using the oligo-nucleotides oHB312 (5’‑TAAAGCGAATTGGATTGCGGCCGCGAATTCATATGGTGAGCAAGG-GCGAGGA‑3’) and oHB313 (5’‑GGATCGAAGCTTGGGCTGCAGGTCGACTCTA-GACTAGATGGTCAGGGTGCCGAACT‑3’). The backbone pMZ333 was amplified using the oligonucleotides oHB308F (5’‑AGTCGACCTGCAGCCCAAGC‑3’) and oHB308R (5’‑GCAAT-TCGATCCGGGACCTGAAAT‑3’). | This work |
| pHB355 | **P_T7_::mEGFP(Y66H)**  Bacterial expression vector encoding mEGFP with the Y66H substitution for blue fluorescence.  pHB004 was PCR amplified using the primers oHB318 (5’‑GTGACCACCCTGACCCATGGCG-‑3’) and oHB319 (5’‑AAGCACTGCACGCCATGGGTCA‑3’) thereby introducing the Y66H substitution resulting in a hexahistidine-tagged blue fluorescent protein. | This work |
| pHB357 | **P_SV40_::mEGFP‑(GS)_12_‑Zip(+)‑pA**  P_SV40_-driven mammalian expression vector encoding the monomeric green fluorescent protein mEGFP coupled to a leucine zipper (Zip+) with a (GS)_12_ peptide linker.  Plasmid pHB080 excluding the Lck sequence was PCR amplified using the oligonucleotides oHB324 (5’‑CGGATCGAATTGCGGCCGCAGGAGGCGCCACCATGGTGAGCAAGGGCGAGG-A‑3’) and oHB325 (5’‑CCGGTGAACAGCTCCTCGCCCTTGCTCACCATGGTGGCGCCTCCT-GCG‑3’). | This work |
| pHB358 | **P_SV40_::MTS‑mEGFP‑(GS)_12_‑NZ‑pA**  SV40 promoter-driven mammalian expression vector encoding the monomeric enhanced green fluorescent protein GFP carrining an aminoterminal mitochondrial targeting signal (MTS) coupled to a leucine zipper (Zip+) with a (GS)_12_ linker.  The whole plasmid pHB357 was PCR amplified using the primers oHB326 (5’‑CGGGGCTTGAC-AGGCTCGGCCCGGCGGCTCCCAGTGCCGCGCGCCAAGATCCATTCGTTGATGGTGAGCA-AGGGCGAGGA‑3’) and oHB327 (5’‑GGGAGCCGCCGGGCCGAGCCTGTCAAGCCCCGCAGC-AGCAGCGGCGTCAGGACGGACATGGTGGCGCCTCCTGCG‑3’) thereby inserting a mito-chondrial targeting signal (MTS, MSVLTPLLLRGLTGSARRLPVPRAKIHSL, amino acid sequence) encoded in the 5’ oligonucleotide extension. | This work |
| pHB368 | **P_SV40_::COP1‑VP16‑NLS-IRES-TetR-UVR8-pA**  SV40 promoter-driven mammalian expression vector encoding the transactivator COP1‑VP16 and the DNA-binding protein TetR-UVR8 for UV light controlled induction of tet-responsive promoters. COP1 was PCR amplified from pKM115 using the oligonucleotides oHB368 (5’‑TTTT-TGTCTTTTATTTCAGGTCCCGGATCGAATTGCGGCCGCAGGAGGCGCCACCATGTATAGCA-ACGGCCTTGCA‑3’) and oHB369 (5’‑GTACGCGCGCGGCTGTACGCGGAACCAGCACTGGC-GCCCGCAGCG‑3’), VP16 was PCR amplified from pKM022 using the oligonucleotides oHB370 (5’‑AGTGCTGGTTCCGCGTACAGCCGC‑3’) and oHB371 (5’‑TCACACCTTCCGCTTTTTCTTG-G‑3’), the IRES sequence was PCR amplified from pKM022 using the oligonucleotides oHB372 (5’‑ACGGTGGGCCCAAGAAAAAGCGGAAGGTGTGATCTAGAGTCGAC-CTGCAGCC‑3’) and oHB373 (5’‑TTAATCACTTTACTTTTATCTAATCTAGACATATGAATTCG-CGGCCGCAATCC‑3’), TetR was PCR amplified from pKM022 using the oligonucleotides oHB374 (5’‑ATGTCTAGATTA-GATAAAAGTAAAGTGATTAACAGC‑3’) and oHB375 (5’‑ATAAGAACCTTACGAGGAGGAGCG-GCGCCGGCACCAGCACTACCAGCACTATCGAGCGCG‑3’) and UVR8 was PCR amplified from pKM168 using the oligonucleotides oHB376 (5’‑GCCGGCGCCGCTCCT‑3’) and oHB377 (5’‑TCATGTCTGGATCGAAGCTTGGGCTGCAGGTCGACTCTAGATTATCCATCGACGCTGAG-TGC‑3’). | This work |
| pHB369 | **P_T7_::mCherry**  T7 promoter-driven bacterial expression plasmid for the red fluorescent protein mCherry.  The expression vector pCDFDuet was amplified by PCR in two parts with 32 bp of shared homology within the resistance cassette using the primer pairs oHB378 (5’-GGACGAGCTGTAC-AAGGGTACCCTCGAGTCTGGTAAAGAAAC-3’) and oHB379 (5’-CGCGAACTGCAATTTGGA-GAATGG-3’) for the first part and oHB380 (5’-TTGCGCTGCCATTCTCCAAATTG-3’) and oHB381 (5’-CCTCGCCCTTGCTCACCGATATCCAATTGAGATCTGCCATATGTATATCTC-3’) for the second part. mCherry was amplified from pHB353 using the primers oHB382 (5’- TCTCAATTGGATATCGGTGAGCAAGGGCGAGGAGGATAAC-3’) and oHB383 (5’- CAGACTC-GAGGGTACCCTTGTACAGCTCGTCCATGCCG-3’). | This work |
| pHB370 | **P_35S_::Renilla-2A-IAA31-Firefly-pA**  35S promoter-driven plant expression vector for the IAA31-based auxin sensor.  Renilla luciferase fused to the 2A peptide was amplified from pSW401 using the oligonucleotides oHB384 (5’-GCTACCGGTCGCCACCATGACTTCGAAAGTTTATGATCCAGAACAAAGG-3’) and oHB385 (5’-GGGTCCAGGATTTGATTCCACGTCGCCG-3’). The pBS 35S:EGFP+term vector was amplified using the oligonucleotides oHB386 (5’- CGGAAAGATCGCCGTGTAACTGATCTCGAGGCGAAT-TTCCC-3’) and oHB387 (5’-AAACTTTCGAAGTCATGGTGGCGACCGGTAGCGCTAGAGTCCC-3’). The IAA31 sensor module fused to the Firefly luciferase was amplified from pSW401 using the oligonucleotides oHB388 (5’-TGGC-CGGCGACGTGGAATCAAATCCTGGAC-3’) and oHB389 (5’- GCCTCGAGATCAGTTACACGG-CGATCTTTCCGCCC-3’). | This work |
| pHB372 | **P_35S_::Renilla-2A-IAA17-Firefly-pA**  35S promoter-driven plant expression vector for the IAA17-based auxin sensor.  Renilla luciferase fused to the 2A peptide was amplified from pSW401 using the oligonucleotides oHB384 (5’-GCTACCGGTCGCCACCATGACTTCGAAAGTTTATGATCCAGAACAAAGG-3’) and oHB385 (5’-GGGTCCAGGATTTGATTCCACGTCGCCG-3’). The pBS 35S:EGFP+term vector was amplified using the oligonucleotides oHB386 (5’-CGGAAAGATCGCCGTGTAACTGATCT-CGAGGCGAAT-TTCCC-3’) and oHB387 (5’-AAACTTTCGAAGTCATGGTGGCGACCGGTAGC-GCTAGAGTCCC-3’). The IAA17 sensor module fused to the Firefly luciferase was amplified from pMK106 using the oligonucleotides oHB388 (5’-TGGC-CGGCGACGTGGAATCAAATCCTGGAC-3’) and oHB389 (5’-GCCTCGAGATCAGTTACACGG-CGATCTTTCCGCCC-3’). | This work |
| pHB373 | **P_35S_::Renilla-2A-Ctrl-Firefly-pA**  35S promoter-driven plant expression vector for the control auxin sensor, encoding an auxin independent (GS)_7_ linker sequence.  Renilla luciferase fused to the 2A peptide was amplified from pSW401 using the oligonucleotides oHB384 (5’-GCTACCGGTCGCCACCATGACTTCGAAAGTTTATGATCCAGAACAAAGG-3’) and oHB385 (5’-GGGTCCAGGATTTGATTCCACGTCGCCG-3’). The pBS 35S:EGFP+term vector was amplified using the oligonucleotides oHB386 (5’-CGGAAAGATCGCCGTGTAACTGATCTC-GAGGCGAATTTCCC-3’) and oHB387 (5’-AAACTTTCGAAGTCATGGTGGCGACCGGTAGCG-CTAGAGTCCC-3’). The control sensor module fused to the Firefly luciferase was amplified from pMZ030 using the oligonucleotides oHB388 (5’-TGGC-CGGCGACGTGGAATCAAATCCTGGAC-3’) and oHB389 (5’- GCCTCGAGATCAGTTACACGG-CGATCTTTCCGCCC-3’). | This work |
| pKM006 | **tetO_13_-488bp-P_CMVmin_::SEAP-pA**  Vector encoding SEAP under the control of a modified P_Tet_, harboring a 422-bp spacer between the 13mer tetO operator and the minimal promoter. | Müller *et al.*[2] |
| pKM022 | **P_SV40_::PhyB(1-650)-VP16-NLS-IRES-TetR-PIF6(1-100)-HA-pA**  Mammalian bicistronic vector encoding PhyB(1-650)-VP16-NLS and TetR-PIF6(1-100)-HA under control of P_SV40_. | Müller *et al.*[2] |
| pKM115 | **P_SV40_::COP1(WD40)-VP16-pA**  Vector encoding COP1(WD40)-VP16 under control of P_SV40_. | Müller *et al.*[3] |
| pKM168 | **P_SV40_::E-UVR8(12-381)-pA**  Mammalian expression vector encoding E-UVR8(12–381) under control of P_SV40_. | Müller *et al.*[3] |
| pMH023 | A bacterial expression vector encoding the *E. coli* codon optimized amino-terminal 100 amino acids of the *Arabidopsis thaliana* Phytochrome Interacting Factor 6 (PIF6). | unpublished |
| pMK106 | EF1α promoter-driven mammalian expression vector encoding the IAA17 auxin sensor. | Wend *et al.*[4] |
| pMZ030 | EF1α promoter-driven mammalian expression vector encoding the control auxin sensor. | Wend *et al.*[4] |
| pMZ333 | P_SV40_-driven mammalian expression vector derived from *Xba*I/*Not*I digested pSAM200 [5]. | unpublished |
| pMZ701 | Plasmid encoding the monomeric red fluorescent protein mCherry. | unpublished |
| pMZ725 | Plasmid encoding the green fliorescent protein mEGFP with the monomerizing point mutation K207A which was originally amplified from pGEMHE-XfA4-mEGFP (kindly provided by M. Ulbrich, Freiburg). | unpublished |
| pSJ027 | A mammalian expression vector encoding the red fluorescent protein mCherry fused to a nuclear localization sequence (NLS, PKKKRKV, amino acid sequence). | unpublished |
| pSW209 | P_35S_-driven plat expression vector encoding Renilla and Firefly. | unpublished |
| pSW401 | EF1α promoter-driven mammalian expression vector encoding the IAA31 auxin sensor. | Wend *et al.*[4] |
| pWW301 | **P_T7_::ET1-His6**  A pRSET-derived bacterial expression vector for the macrolide-responsive transactivator ET1. | Weber *et al.*[6] |

**Abbreviations:** Avitag, target biotinylation signal for the bacterial biotin-ligase BirA; COP1(WD40), WD40 domain of CONSTITUTIVELY PHOTOMORPHOGENIC 1 from *Arabidopsis thaliana*; E, macrolide-responsive repressor protein; EF1α, translation elongation factor EF1α, ET1, tagged macrolide-responsive transactivator; HA, epitope tag derived from the human influenza hemagglutinin protein; IRES, polioviral internal ribosome entry site; Lck, lymphocyte-specific protein tyrosine kinase; MTS, mitochondrial targeting signal; NES, nuclear export sequence derived from the minute virus of mice; NLS, nuclear localization sequence from the simian virus 40 large T antigen; P_35S_, Cauliflower Mosaic Virus 35S promoter; PIF6, Phytochrome Interacting Factor 6; PhyB, Arabidopsis thaliana photoreceptor protein Phytochrome B; P_SV40_, simian virus 40 early promoter; SEAP, human placental secreted alkaline phosphatase; tetO, TetR-binding operator sequence; TetR, *E. coli* derived tetracycline repressor protein; UVR8, *Arabidopsis thaliana* UV-resistance locus 8 photoreceptor protein; VP16, *Herpes simplex* derived transcriptional transactivation domain; Zip(+), leucine zipper element.

**Supplementary Table B. AQUA Cloning conditions of PCR derived DNA fragments. Numbers of colonies derived from AQUA Cloning employing a PCR derived vector and insert DNA fragment (pos.), or the vector fragment only (neg.) at the thermal conditions and with the lengths of homologies indicated. The accuracy for each condition was determined by analytical colony PCR of eight clones using the oligonucleotides oHB304 and oMZ558. Abbreviation: acc., accurancy; neg., number of PCR negative clones; pos., number of PCR positive clones; RT, room temperature.**

|  |  | ice | | |  | RT | | |  | 50 °C | | |
| --- | --- | --- | --- | --- | --- | --- | --- | --- | --- | --- | --- | --- |
| homology |  | pos. | neg. | acc. |  | pos. | neg. | acc. |  | pos. | neg. | acc. |
| 16 bp |  | 1140 | 17 | 8/8 |  | 1480 | 21 | 8/8 |  | 580 | 16 | 8/8 |
| 24 bp |  | 1800 | 17 | 8/8 |  | 1780 | 21 | 8/8 |  | 600 | 16 | 7/8 |
| 32 bp |  | 1300 | 17 | 8/8 |  | 1980 | 21 | 8/8 |  | 1120 | 16 | 8/8 |


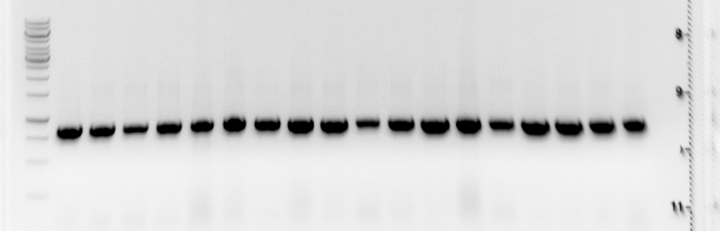

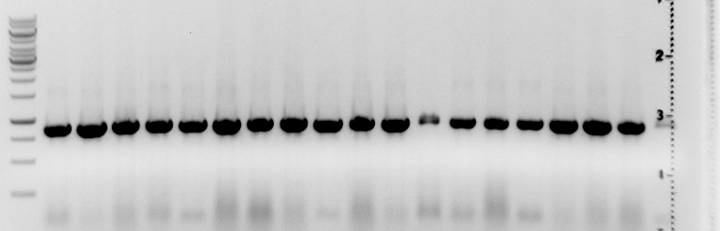


16 bp homology

24 bp

homology

32 bp homology

RT

RT

1 2 3 4 5 6 7 8

1 2 3 4

5 6 7 8

1 2 3 4 5 6 7 8

**Supplementary Fig A. Analytical colony PCR for optimized AQUA Cloning conditions.** Analytical colony PCR of clones obtained from AQUA Cloning with PCR derived DNA and with incubation at room temperature (RT). Results are shown for homologies of 16 bp, 24 bp and 32 bp. PCRs were performed using the oligonucleotides oHB304 and oMZ558.


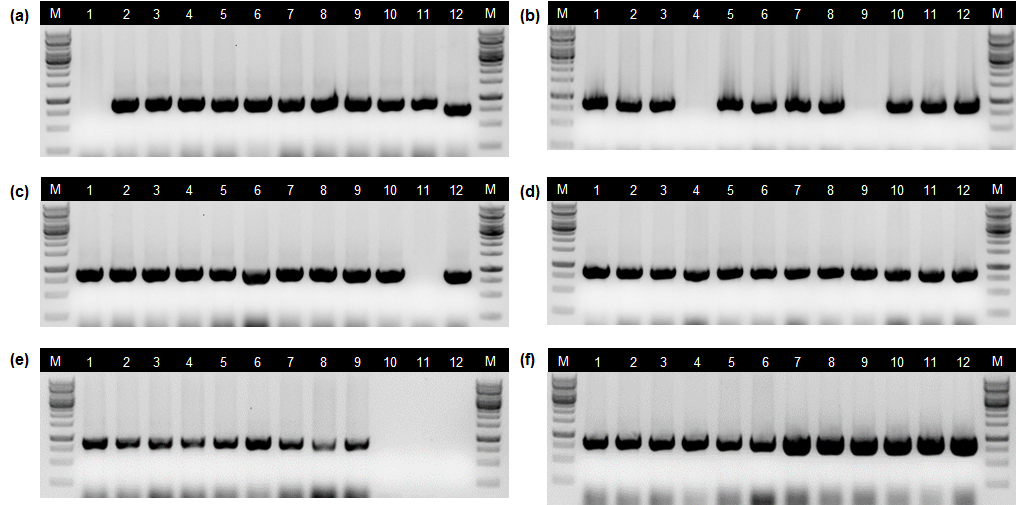


**Supplementary Fig B. Efficiency of the 2-fragment assembly in different common strains of *E. coli*.** Analytical colony PCR of 12 clones of each *E. coli* strain (BL21 (DE3), 9 clones) was performed using the oligonucleotides oMZ558 and oHB304. **(a)** TOP10, home-made. **(b)** TOP10, Invitrogen, **(c)** NEB5α, NEB. **(d)** NEB10β, NEB. **(e)** BL21 (DE3), NEB. **(f)** JM109, Promega.

**
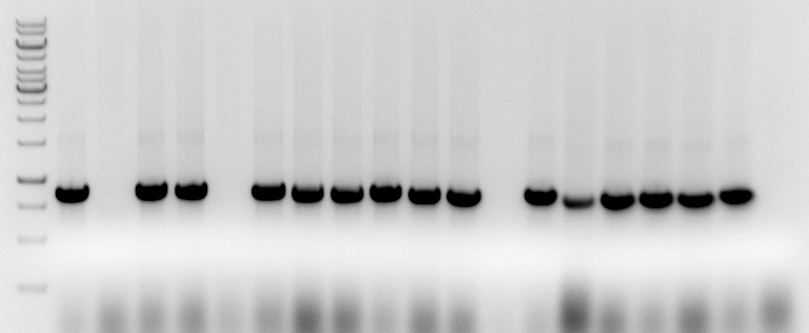
**

1

2

3

4

5

6

7

8

9

10

11

12

13

14

15

16

17

18

neg.

M

**Supplementary Fig C. Efficiency of the 4-fragment *de novo* assembly.** Analytical colony PCR of 18 clones (out of 360 colonies) was performed using the oligonucleotides oMZ558 and oHB304. A single colony (out of 7) from a negative condition with the vector fragment only was used as a negative control (neg).

**References**

1. Yan H, Marquardt K, Indorf M, Jutt D, Kircher S, Neuhaus G, et al. Nuclear Localization and Interaction with COP1 Are Required for STO/BBX24 Function during Photomorphogenesis. Plant Physiol. 2011;156: 1772–1782.

2. Müller K, Engesser R, Metzger S, Schulz S, Kämpf MM, Busacker M, et al. A red/far-red light-responsive bi-stable toggle switch to control gene expression in mammalian cells. Nucleic Acids Res. 2013;41: e77.

3. Müller K, Engesser R, Schulz S, Steinberg T, Tomakidi P, Weber CC, et al. Multi-chromatic control of mammalian gene expression and signaling. Nucleic Acids Res. 2013;41: e124.

4. Wend S, Dal Bosco C, Kämpf MM, Ren F, Palme K, Weber W, et al. A quantitative ratiometric sensor for time-resolved analysis of auxin dynamics. Sci Rep. 2013;3: 2052.

5. Fussenegger M, Moser S, Mazur X, Bailey JE. Autoregulated multicistronic expression vectors provide one-step cloning of regulated product gene expression in mammalian cells. Biotechnol Prog. 1997;13: 733–740.

6. Weber CC, Link N, Fux C, Zisch AH, Weber W, Fussenegger M. Broad-spectrum protein biosensors for class-specific detection of antibiotics. Biotechnol Bioeng. 2005;89: 9–17.
